# Direct Detection of Viruses Using Antibody‐Modified Gold Nanorods

**DOI:** 10.1002/jbio.202500417

**Published:** 2025-10-28

**Authors:** Axell Rodriguez, Yana Purvinsh, Aidan P. Holman, Dmitry Kurouski

**Affiliations:** ^1^ Department of Biochemistry and Biophysics Texas A&M University College Station Texas USA; ^2^ Interdisciplinary Faculty of Toxicology Texas A&M University College Station Texas USA

**Keywords:** dynamic light scattering, gold nanorods, machine learning, viruses

## Abstract

Seasonal and sporadic viral infections put enormous burdens on global health and society. Although polymerase chain reaction (PCR) and other molecular approaches can be used to detect and identify viruses, they require substantial sample processing time and often have limited portability, limiting their utility in point‐of‐care settings. In the current study, we investigate the accuracy of direct sensing of two bacteriophages using chemically modified gold nanorods (AuNRs). We demonstrate that when using functionalized polyethylene glycol (PEG), the surface of AuNRs can be modified with desired antibodies against a particular pathogen. Furthermore, PEG protects and passivates the surface of AuNRs against unspecific binding of biomolecules that can be present in the body fluids. We also showed that dynamic light scattering (DLS) was capable of detecting virus‐bound AuNRs, enabling confirmatory detection of viruses. These results indicate that antibody‐modified AuNRs can be used for the confirmatory detection of various viruses.

## Graphical abstract

Gold nanorods functionalized with pathogen‐specific antibodies can be used for the confirmatory detection of viruses via dynamic light scattering–based analysis of particle sizes.

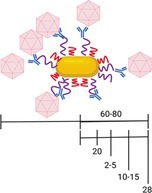

## 1 Introduction

H1N1 subtype of the influenza A virus, also known as Spanish flu, and Severe Acute Respiratory Syndrome Coronavirus 2 (SARS‐CoV‐2) caused millions of deaths in the past years [1, 2, 3]. While some viruses are deadly for humans, others pose a serious threat to global food security [4, 5, 6]. For instance, African swine fever (ASF) is a contagious and deadly viral swine disease that affect domestic and feral swine of all ages [7, 8, 9, 10]. H5 bird flu is widespread in wild birds worldwide that cause outbreaks in poultry and United States which results in detrimental losses to US poultry [11].

Polymerase chain reaction (PCR) is one of the most commonly used molecular techniques for viral detection and identification [12]. This approach requires primers against the targeted virus and often has difficulty in distinguishing closely related viral species [12]. PCR is also sensitive to organic contaminants that may cause both false negative and false positive results [13]. Some of these issues can be overcome by Matrix‐Assisted Laser Desorption/Ionization Time‐of‐Flight Mass Spectrometry (MALDI‐TOF MS) [14]. This technique is based on viral identification based on the unique proteins present in the pathogens [15]. However, MALDI‐TOF MS has limited portability and requires highly expensive instruments, which are typically absent in most clinics and diagnostic laboratories [16].

To overcome these limitations, numerous nanoparticle and membrane‐based aptamer‐, enzyme‐ and immunoassays were developed over the past decade [17, 18, 19]. For instance, Layqah and coworkers developed a nano‐immunosensor specific to spike protein S1 in MERS‐CoV [20]. The sensor used carbon electrodes that were functionalized using AuNPs. The researchers showed that AuNPs‐based nano‐immunosensor provided MERS detection within 0.001 and 100 ng/mL displaying an enhanced sensitivity of 0.4 pg/mL [20]. Seo and colleagues developed a graphene‐based sensor that could detect SARS‐CoV‐2 viral particles. In this sensor, graphene was modified with SARS‐CoV‐2 spike antibodies [21]. The sensor enabled detection of viral particles at 1 fg/mL. Such sensing is typically based on colorimetric, electrochemical or photoelectrochemical assays or optical shifts of nanoparticle absorption spectra, also known as localized surface plasmon resonances (LSPRs) [17].

In the current study, we investigate the feasibility of using dynamic light scattering (DLS) to detect antibody‐modified nanoparticle‐virus binding. This approach was previously successfully used to detect influenza and SARS‐CoV‐2 viruses [22, 23]. For this, gold nanorods (AuNRs) developed in our laboratory were modified using a mixture of carboxy (CT) and methoxy (MT) thiolated polyethylene glycol (CT/MT‐PEG). Ct‐PEG enabled covalent binding of desired antibodies to the nanostructures, while MT‐PEG served as a spacer for CT‐PEG. Both CT and MT PEGs also protected antibodies against charged‐induced conformational changes that could be triggered by AuNPs, as well as protect the nanostructures against unspecific binding of biomolecules that can be present in the body fluids.

## 2 Materials and Methods

### 2.1 Materials

Gold(III) chloride hydrate (HAuCl_4_), 254 169, N‐hydroxysuccinimide (NHS), 130 672, silver nitrate (AgNO_3_), 7761‐88‐8, L‐ascorbic acid, A5960, hexadecyltrimethylammonium bromide (CTAB), H6269, sodium borohydride (NaBH_4_), 71 320, Antienterobacterio Phage MS2 Coat Protein, ABE76‐I, ammonium sulfate ((NH_4_)_2_SO_4_), A4418 and tetracycline hydrochloride, T7660 were purchased from Sigma‐Aldrich (St. Lois, MO, USA). MT(PEG)4 Methyl‐PEG‐Thiol Compound (MT‐PEG), 26 132, CT(PEG)12 Carboxy‐PEG‐Thiol Compound (CT‐PEG), 26 133, 1‐Ethyl‐3‐[3‐dimethylaminopropyl]carbodiimide hydrochloride (EDC), 22 980, MES Buffered Saline, 28 390, Alpha Synuclein Recombinant Mouse Monoclonal Antibody (10E5), MA5‐50223, Escherichia Virus Capsid Polyclonal Antibody, PA5‐144462, and Goat antimouse IgG (H + L) Secondary Antibody, DyLight 633, 35 512 were purchased from Thermo Fisher Scientific (Hoston, TX, USA). Cesium Chloride, BP210 (CsCl) was purchased from Fisher Scientific (Hampton, NH, USA). Antimouse IgG (H + L)—20 nm Gold Conjugate, AC‐20‐02‐05 was purchased from Cytodiagnostics (Burlington, ON, Canada).

### 2.2 Synthesis of Gold Nanoparticles (AuNPs)

First, 250 μL of HAuCl_4_ (0.01 M) in 9.75 mL of CTAB (0.1 M) was mixed with 600 μL ice‐cold NaBH_4_ (0.001 M) to prepare seeds. Once mixed, the solution was left at room temperature for 1 h. The color of the solution immediately changed from yellow to colorless and then became amber in 1 h. Next, fresh seeds were used to fabricate AuNRs. For this, 48 μL of the fresh seed solution was mixed with a premade solution of 80 μL AgNO_3_ (0.01 M), 38 mL CTAB, 2 mL HAgCl_4_, and 220 mL of ascorbic acid (0.1 M). The solution was left for 2 h at 35°C with constant stirring. To remove CTAB, AuNRs were centrifuged at 11 000 rcf for 30 min. Next, the formed pellet was resuspended in 10 mL of DI water.

### 2.3 PEG Coating of AuNPs

Prior to PEG coating, AuNRs were centrifuged at 11 000 rcf for 30 min to remove residual CTAB present in the solution. To prepare CT/MT‐PEG AuNRs, 0.4 mM MT‐PEG and 1.6 mM CT‐PEG were first dissolved in DMSO and then added to 1 mL of AuNRs in Tris‐carbonate buffer (pH 3.0). To prepare CT‐PEG coated AuNRs, 2 mM CT‐PEG was dissolved in DMSO and then mixed with the same volume of AuNRs, as was described above. Next, the solution was left for 30 min under constant agitation (180 rpm) and finally centrifuged at 11 000 rcf for 30 min.

### 2.4 Antibody‐Conjugation

CT/MT‐PEG‐coated AuNRs were first resuspended in 1 mL MES buffer (0.1 M, pH 6.0). Next, 40 μg of 1‐ethyl‐3‐(3‐dimethylaminopropyl)carbodiimide hydrochloride (EDC) and 100 μg of NHS were added to AuNRs and kept shaking at 180 rpm for 15 min at room temperature. The solution was centrifuged at 11 000 rcf for 30 min to remove unreacted reagents. A formed pellet that contained CT/MT‐PEG‐coated AuNRs was resuspended in 500 μL of phosphate buffer saline (PBS), pH 7.4. Next, 3 μg of antibodies (Ab) to CT/MT‐PEG‐coated AuNRs were incubated at 180 rpm for 2 h at room temperature. To quench unreacted NHS, 5 μL of 1 M hydroxylamine was added and kept at 180 rpm for 10 min. The solution was centrifuged at 11 000 rcf for 30 min to remove unreacted reagents.

For fluorescence microscopy (FM) imaging, secondary Ab labeled with a red (DyLight 633) fluorophore was added in a ratio of 1:5000 v/v. For TEM imaging, secondary Ab labeled with gold nanospheres were added in a ratio 1:100 v/v.

### 2.5 Qbeta and MS2 Expression and Purification

*Escherichia coli* (ER2738) was transformed with plasmids encoding Qbeta and MS2 viruses. ER2738 was cultured in 5 mL LB with 10 μg/mL of tetracycline for 16 h. A phage titer assay was performed to identify the location of single‐phage plaques that were used in subsequent experiments. A small fraction of the single‐phage plaque was transferred using a pipette tip into 5 mL of LB that contained ER2738 with 10 μg/mL of tetracycline. LB was incubated at 37°C for 8 h with subsequent centrifugation to remove the bacterial‐rich pellet. The supernatant was harvested and filtrated using a 0.2 μm filter.

ER2738 bacterial host cell in 200 mL of LB with 10 μg/mL of tetracycline was incubated at 37°C under 200 rpm stirring until OD600 reached 0.5. Next, the supernatant obtained at the previous experimental stage was added to the bacterial culture and incubated together for 8 h. Finally, the bacterial culture was centrifuged. A formed pellet that mostly contained bacterial cells was discarded. The supernatant was harvested and filtered using a 0.2 μm filter.

For purification of Qbeta and MS2 viruses, (NH_4_)_2_SO_4_ was used. For this, 56 g of (NH_4_)_2_SO_4_ was slowly added to the phage‐enriched supernatant and kept at 4°C under constant stirring overnight. The solution was then centrifuged at 15 000 rcf for 1 h. The pellet was resuspended in 10 mL of 50 mM Tris buffer pH 8.0 containing 150 mM NaCl and 5 mM EDTA and dialyzed against the same buffer for 8 h to remove residual (NH_4_)_2_SO_4_. Finally, CsCl ultracentrifugation was performed to concentrate and isolate pure Qbeta and MS2 viruses with a subsequent dialysis against 50 mM Tris buffer pH 8.0 containing 150 mM NaCl and 5 mM EDTA to remove residual CsCl.

### 2.6 DLS

For the DLS sensing, 1 × 10^9^ phage‐forming units were mixed with functionalized AuNRs. DLS readings were performed using Wyatt DynaPro NanoStar in a quartz cuvette.

## 3 Results and Discussion

**FIGURE 1:**
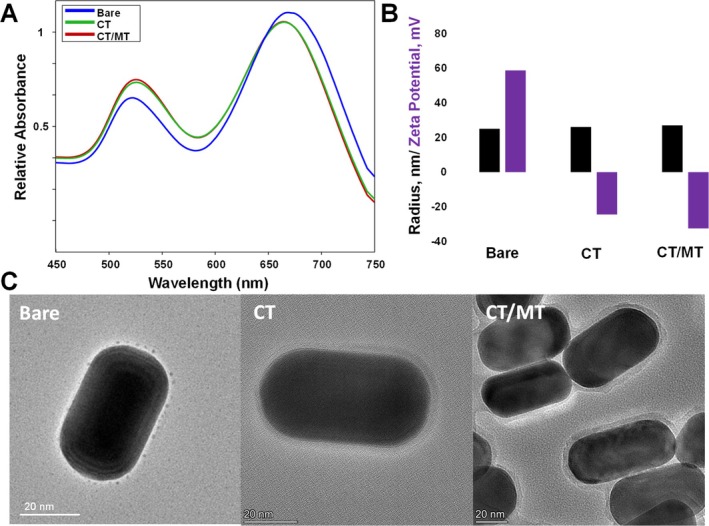
Absorbance spectra (A), DLS and Z potential (B), and TEM (C) of bare, CT‐coated, and CT/MT‐coated AuNPs.

Gold nanorods (AuNRs) were synthesized using experimental procedures reported by the Murphy group [24]. Specifically, gold (III) chloride was reduced by ascorbic acid in the presence of cetyltrimethylammonium bromide (CTAB), silver nitrate, and gold seeds. Silver nitrate facilitated longitudinal growth of gold seeds, while CTAB retained the developed AuNRs in solution. Once synthesized, AuNRs were characterized using absorbance spectroscopy, DLS and transmission electron microscopy (TEM), Figure 1. Next, freshly synthesized AuNRs were centrifuged to remove the excess of CTAB and coated with CT and CT/MT PEG, which formed a monolayer on their surfaces. UV–Vis spectroscopy revealed a small blue‐shift in the absorbance of AuNPs exposed to both CT and CT/MT PEG, which indicates the change in the dielectric properties of the nanostructures, Figure 1A [25]. These results were confirmed by Zeta potential measurements which detected a drastic change in the surface potential of the nanostructures from 58.7 mV (bare) to −24.5 mV and −32.5 mV as a result of their coating with CT and CT/MT PEGs, respectively, Figure 1B. It should be noted that PEG prevented self‐aggregation of the nanostructures. This conclusion could be made based on DLS analysis of radii of bare, CT, and CT/MT‐PEG coated AuNRs. Finally, TEM analysis of the developed CT‐ and CT/MT‐PEG nanostructures revealed the presence of the uniform PEG layer around their surfaces, Figure 1C. It is important to note that functionalized AuNRs did not exhibit any SERS effect when illuminated with 785 nm laser excitation, while bare AuNRs were SERS active at the same experimental conditions (excitation wavelength and AuNRs concentration).

**FIGURE 2:**
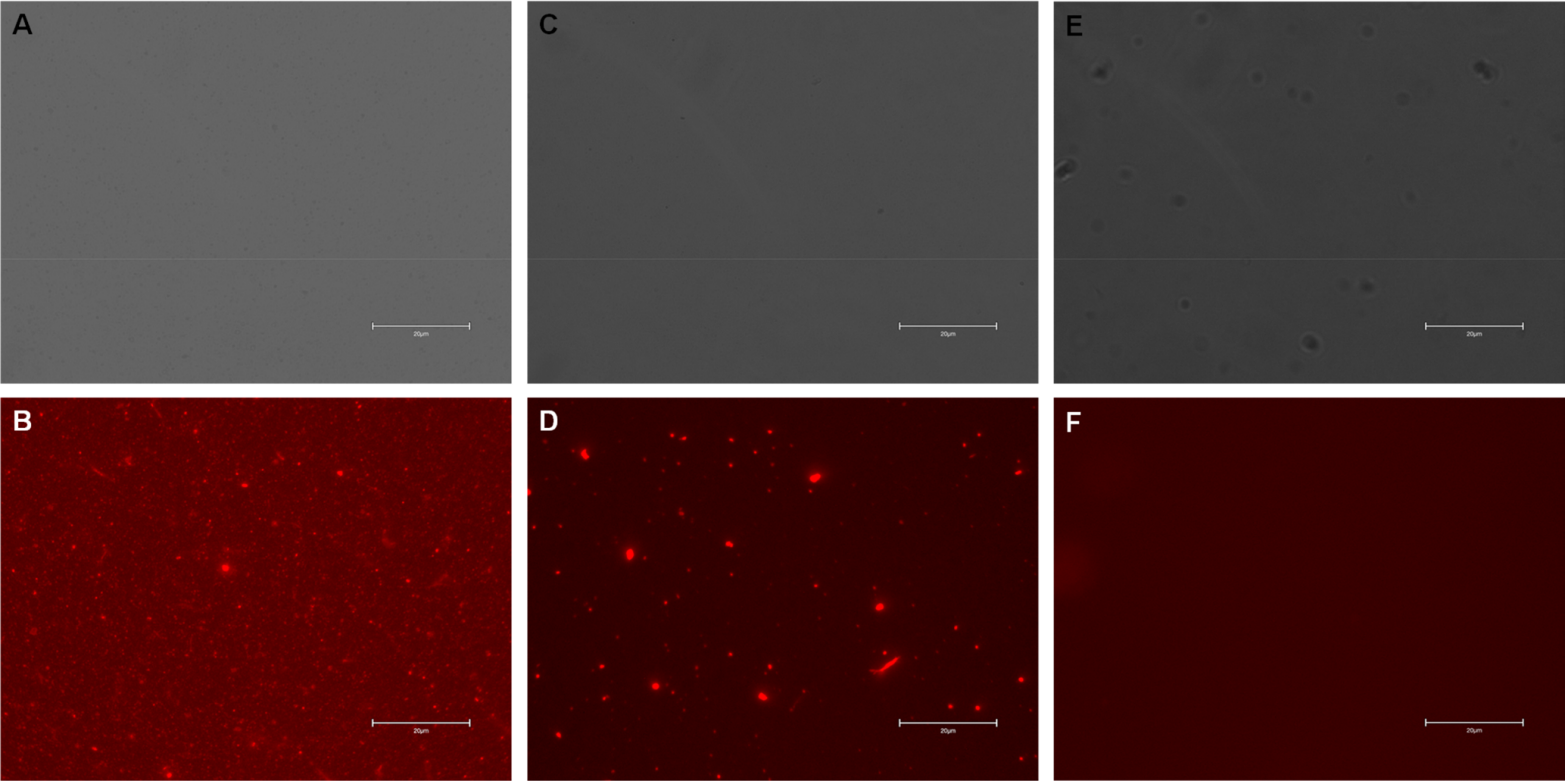
Bright‐field (A, C, and E) and fluorescence (B, D, and F) images of Ab‐AuNPs with the secondary antibody (A and B), AuNPs exposed to Ab and the secondary antibody without EDC and sulfo‐NHS reaction (C and D) and bare AuNRs. Scale bars are 20 μm.

Next, CT/MT PEG‐modified AuNPs were exposed to the antibodies (Ab) against two bacteriophages, MS2 and Qbeta in the presence of EDC and sulfo‐NHS, which enabled covalent attachment of Ab to the nanostructures. We utilized FM to examine functionality of the developed sensor. For this, Ab‐AuNPs were exposed to the secondary Ab labeled with a red (DyLight 633) fluorophore (FM). We observed a strong fluorescence signal which indicates sensor functionality, Figure 2. It should be noted that in the absence of EDC and sulfo‐NHS reaction, fluorescence was observed only in large, clumped particles which indicate physical trapping of Ab in such metallic conglomerates.

**FIGURE 3:**
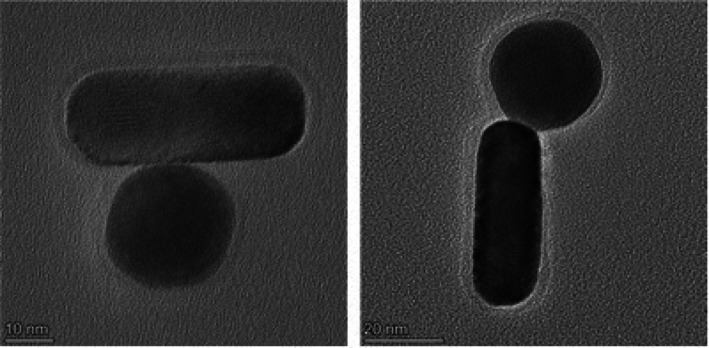
TEM images of Ab‐AuNRs and AuNSs possessing secondary Ab.

Ab‐AuNRs were also exposed to gold nanospheres (AuNSs) coated with the secondary Ab. TEM revealed strong interactions between Ab‐AuNRs and AuNSs possessing secondary Ab, Figure 3. These results indicate that Ab present on the surface of AuNRs are not denatured by the nanostructures themselves and can be used to find the targeted pathogen.

**FIGURE 4:**
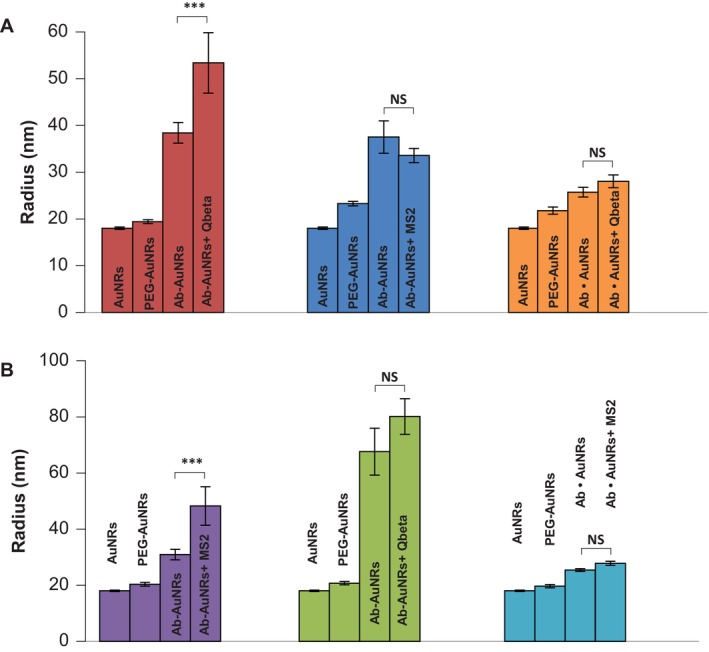
DLS readings from bare AuNRs, CT/MT PEG‐coated AuNRs (PEG‐AuNRs), CT/MT PEG‐coated AuNRs with Ab against Qbeta (A) and MS2 (B) in the absence of the pathogen (Ab‐AuNRs) and in the presence of Qbeta (Ab‐AuNRs + Qbeta) and MS2 (Ab‐AuNRs + MS2). AuNPs exposed to Ab and secondary antibody without EDC and sulfo‐NHS reaction without (Ab • AuNPs) and with corresponding viruses (Ab • AuNPs + Qbeta) and (Ab • AuNPs + MS2). According to T‐ and Welch's‐tests, ***p < 0.001; NS, nonstatistical significance.

Next, Ab‐AuNRs with Qbeta‐ and MS2‐specific Ab were exposed to Qbeta and MS2 viruses. DLS readings were performed to determine changes in the size of the nano sensor, Figure 4. DLS revealed an increase in size of the nanostructures only in the case of the presence of Ab that targeted the virus of interest. Specifically, we observed an increase in the nanostructure size from 40 to 52 nm in the case of Ab‐AuNRs with Ab against Qbeta exposed to the solution with Qbeta. At the same time, no significant increase in the size of Ab‐AuNRs with Ab against Qbeta was observed if the nanostructures were exposed to MS2. The same results were observed for the negative control in which EDC and sulfo‐NHS reaction was not performed to covalently bind Ab to AuNRs (Ab • AuNPs).

DLS also revealed an increase in the nanostructure size from 36 to 52 nm in the case of Ab‐AuNRs with Ab against MS2 exposed to the solution with MS2. We also observed no significant increase in the size of Ab‐AuNRs with Ab against MS2 was observed if the nanostructures were exposed to Qbeta, as well as in the negative control in which EDC and sulfo‐NHS reaction was not performed to covalently bind Ab to AuNRs (Ab • AuNPs).

## 4 Conclusions

Our findings demonstrate that Ab‐modified AuNRs can be used for the direct detection of viruses in aqueous media. We also showed that DLS could be used to quantify the increase in the size of Ab‐modified AuNRs which takes place only in the case of nanostructure‐virus binding. The chemical stability of Ab‐modified AuNRs and the inert metal surface achieved by PEG coating prevents unspecific binding of viruses and excludes false‐positive increase in the size of the nanostructures. Thus, our findings demonstrate that antibody‐modified AuNRs can be used for the rapid, on‐site and confirmatory detection of different viruses in aqueous media.

## Conflicts of Interest

The authors declare no conflicts of interest.

## Data Availability

The data that support the findings of this study are available from the corresponding author upon reasonable request.
